# A resected case of malignant peripheral nerve sheath tumor arising in the cervical esophagus

**DOI:** 10.1186/s44215-023-00082-x

**Published:** 2023-08-03

**Authors:** Eito Nakagawa, Akinori Miura, Kunihito Suzuki, Katsumasa Saito, Hajime Shinohara, Koudai Ueno, Yu Naito, Toru Motoi

**Affiliations:** 1 Department of Esophageal Surgery, Tokyo Metropolitan Cancer and Infectious Diseases Center, Komagome Hospital, 3-18-22 Honkomagome, Bunkyo-Ku, Tokyo, 113-8677 Japan; 2 Department of Pathology, Tokyo Metropolitan Cancer and Infectious Diseases Center, Komagome Hospital, 3-18-22 Honkomagome, Bunkyo-Ku, Tokyo, 113-8677 Japan

**Keywords:** Malignant peripheral nerve sheath tumor (MPNST), Esophagus, Post-irradiation development

## Abstract

We report herein a case of malignant peripheral nerve sheath tumor (MPNST), an extremely rare, esophageal malignancy. A 67-year-old, female patient presented with a nodular lesion in the cervical esophagus which was detected on follow-up computed tomography (CT) after surgery for bilateral breast cancer and gastric cancer. Upper gastrointestinal endoscopy revealed a hemispheric, submucosal lesion in the cervicothoracic esophagus. Endoscopic ultrasound-guided fine needle aspiration cytology revealed spindle-shaped cells with nuclear atypia and positive staining for the S100 protein, a neurogenic marker. Based on clear CT findings of lesion growth over two years, MPNST was diagnosed, and treatment for sarcoma was begun. After one course of preoperative chemotherapy, open resection of the esophagus was performed, revealing a solid, white tumor with a maximum diameter of 1.8 cm. The tumor was histopathologically found to be located within the intrinsic muscularis propria. Based on this finding, low-grade MPNST with a schwannoma-like component at its margins was diagnosed. Approximately 50% of MPNST cases occur against a background of neurofibromatosis type 1 (NF1) while about 40% occur sporadically, and 10% occur after irradiation. The patient’s history of radiotherapy for left breast cancer may have contributed to the development of the MPNST.

## Background

Malignant peripheral nerve sheath tumor (MPNST) is a rare, malignant spindle cell tumor of the peripheral nerves. We report herein a highly unusual case of a gradually increasing, submucosal tumor in the cervical esophagus occurring within an area irradiated 14 years previously following postoperative radiotherapy for breast cancer. Based on an analysis of a biopsy specimen and the clinical course, MPNST was eventually diagnosed.

## Case presentation

**Fig. 1:**
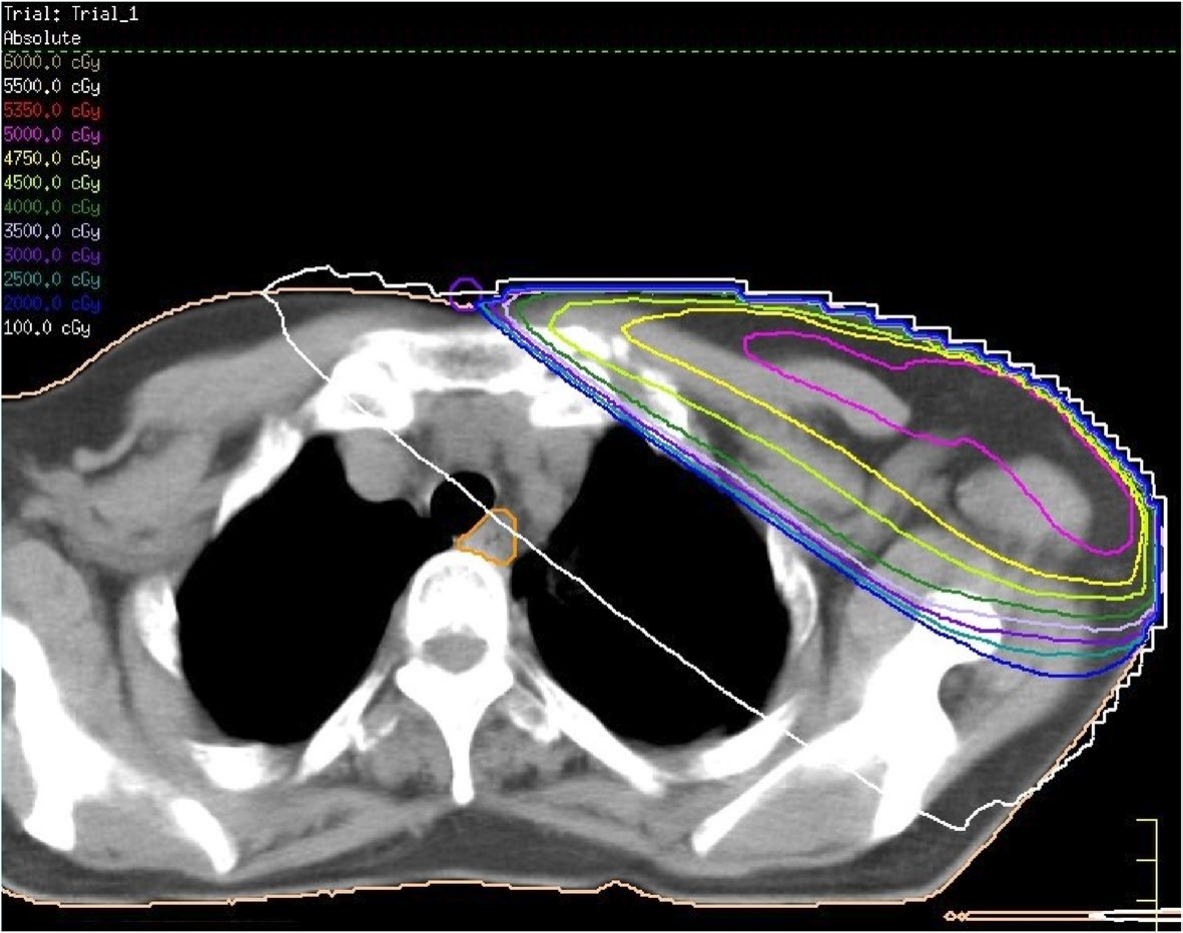
Simulated image of the postoperative radiation field of left breast cancer in this patient

A 67-year-old, female patient receiving regular follow-up examinations after breast cancer treatment visited the study center for a close examination after a mass in the left cervical esophagus was detected on computed tomography (CT). When 42 years old, she received a right total mastectomy for breast cancer; at the age of 51 years, she underwent left breast conservation surgery, postoperative radiation therapy (total breast irradiation 50 Gy/25 fr + boost 10 Gy/5 fr), and postoperative adjuvant chemotherapy with Endoxan, Arimidex, and Fluturon. As a reference, Fig. 1 shows a simulated image of the radiation field of left breast cancer in this patient. At the age of 54 years, she received a laparoscopic pyloric gastrectomy for gastric cancer. Her family history was otherwise unremarkable. Blood analysis revealed elevated tumor markers, CA19-9 and CA125, at 116.9 U/mL and 273.5 U/mL, respectively. No other abnormal findings were noted.

**Fig. 2:**
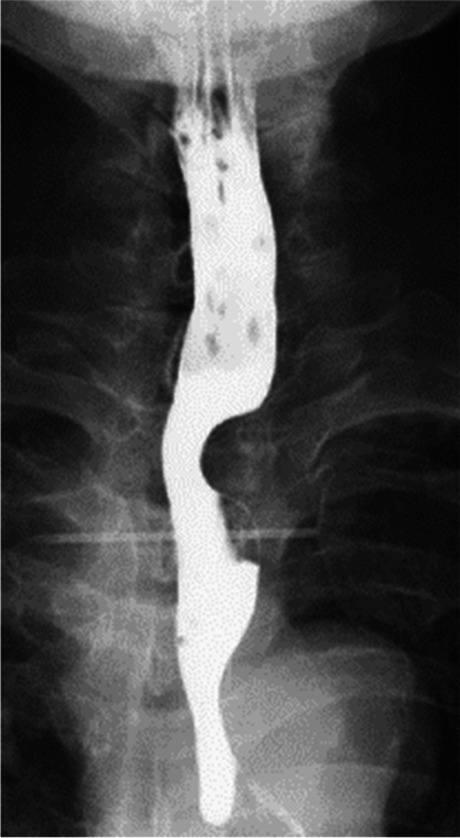
Esophagography findings: The lesion appeared as a shadow defect protruding into the left wall of the cervical esophagus with a steep rise

**Fig. 3:**
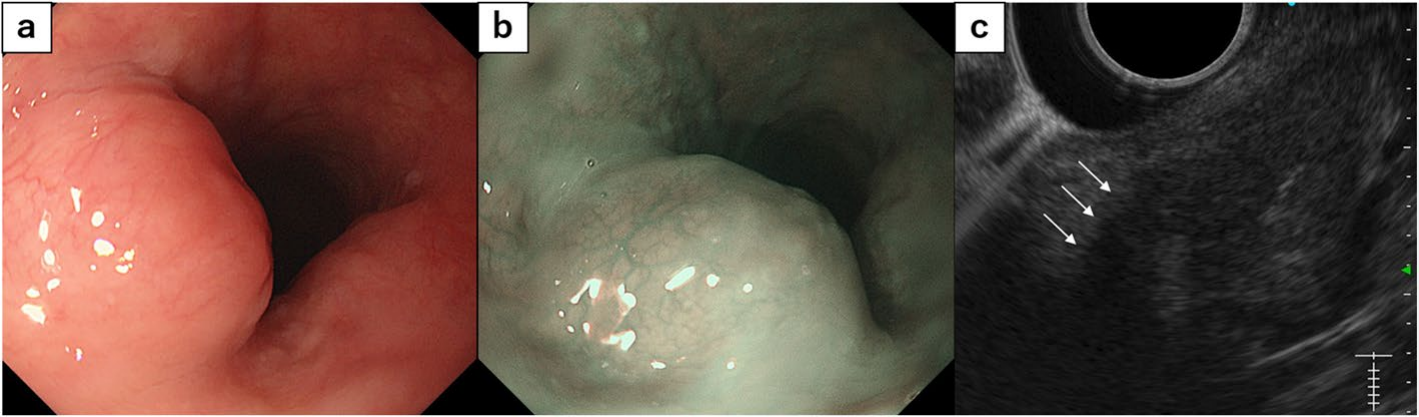
Upper gastrointestinal endoscopic findings. **a** An elevated lesion covered by normal mucosa was observed in the left wall of the cervicothoracic esophagus 16 cm from the incisor. Approximately one-quarter of its circumference was visible. **b** Observation with NBI (narrow-band imaging). **c** Ultrasonographic findings. A hypoechoic area with a maximum diameter of 16 mm was observed at the same site. The lesion was located in the intrinsic muscularis propria. Its interior was heterogeneous, its margins were irregular, and the boundary between the muscular layer and perimural tissue was unclear (white arrows)

Esophagography (Fig. 2) visualized the lesion as a shadow defect protruding into the left wall of the cervical esophagus with a steep rise. Upper gastrointestinal endoscopy (Fig. 3) demonstrated an elevated lesion covered by normal mucosa; approximately one-quarter of its circumference was visible in the left wall of the cervicothoracic esophagus 16 cm from the incisors. Ultrasonography found an hypoechoic area with a maximum diameter of 16 mm at the same site. The lesion was located in the intrinsic muscularis propria; its interior was heterogeneous, its margins were irregular, and the boundary between the muscular layer and the perimural tissue was unclear. Fine needle aspiration (FNA) was performed at the same site for diagnosis.

**Fig. 4:**
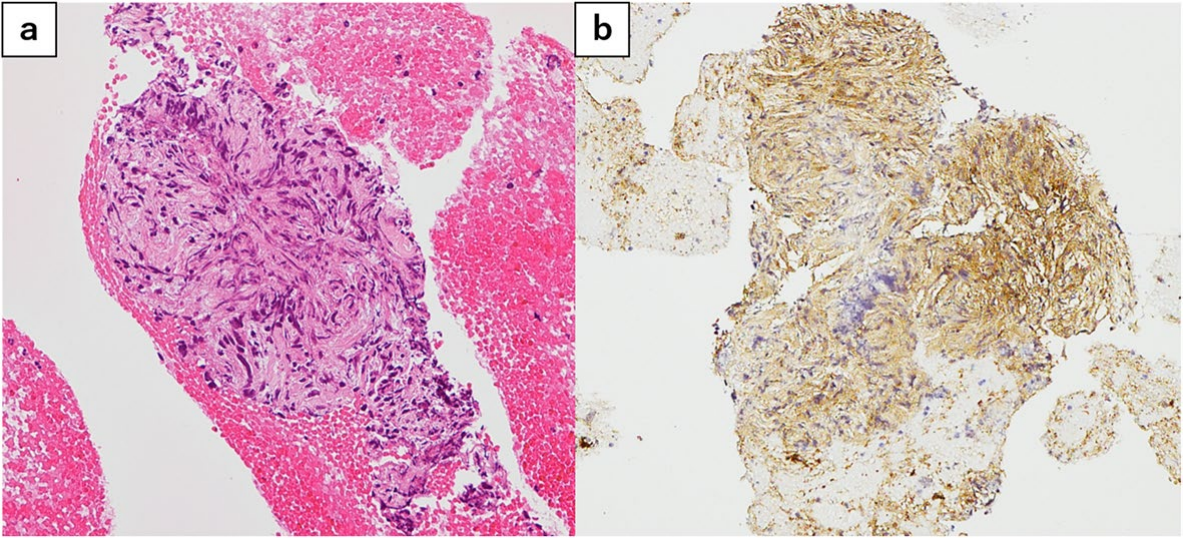
EUS-FNA histopathological findings. **a** H.E. staining, × 200. Spindle-shaped cells with nuclear atypia were observed despite strong contusion. **b** Immunohistochemical staining. Neurogenic marker S100 stained positive

Figure 4 shows the endoscopic ultrasound-guided fine needle aspiration (EUS-FNA) histopathological findings. While the specimen was small and the tissue was highly contused, it clearly contained spindle-shaped cells with nuclear atypia and stained positive for S100 protein, a neurogenic marker. Although it was difficult to determine whether the tumor was benign or malignant, a neurogenic tumor was suspected.

**Fig. 5:**
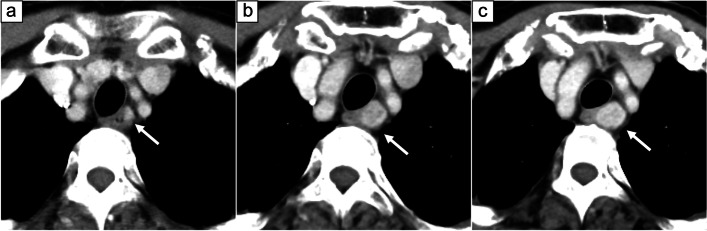
Contrast-enhanced CT findings. An internally heterogeneous, nodular lesion with contrast effect was observed on the left side of the cervical esophagus (white arrow). The maximum diameter of the lesion was **a** 7 mm in July 2019, **b** 14 mm in December 2020, and **c** 17 mm in May 2021, demonstrating rapid growth over about 2 years

**Fig. 6:**
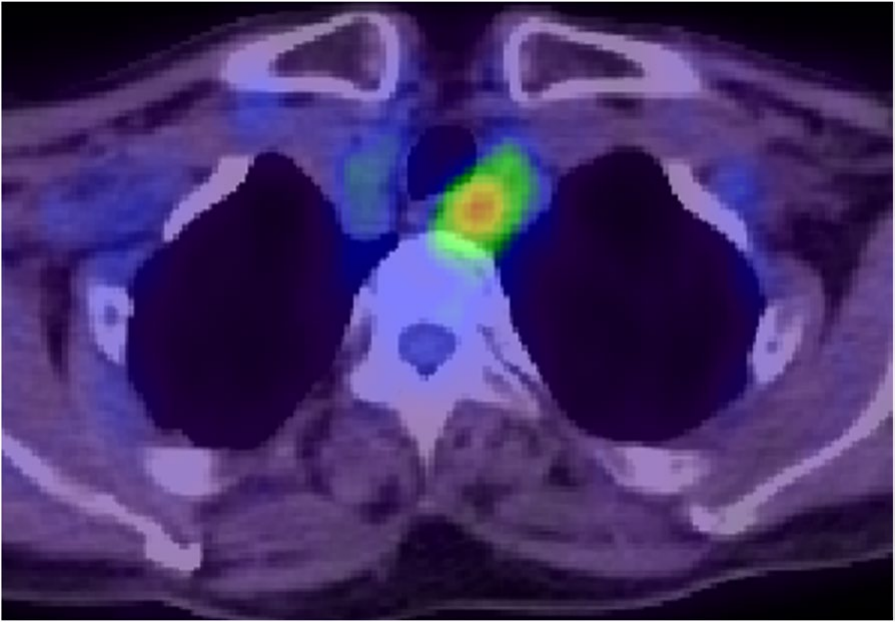
PET-CT findings. The maximum standardized uptake value increased from 3.85 to 4.78 at the same site

Contrast-enhanced CT (Fig. 5) found a 17-mm, internally heterogeneous, nodular lesion with contrast effect on the left cervical esophagus. The lesion clearly increased in diameter over a period of about two years; its maximum diameter in July 2019, December 2020, and May 2021 was 7 mm, 14 mm, and 17 mm, respectively. Positron emission tomography-computed tomography (PET-CT) (Fig. 6) demonstrated an increase in the standardized uptake value from 3.85 to 4.78 in the same site.

Based on the imaging findings, which clearly demonstrated tumor enlargement, and the pathological analysis of the biopsy specimen, malignant peripheral nerve sheath tumor was diagnosed. After consultation with the Department of Bone and Soft Tissue Oncology, the patient began receiving treatment for sarcoma. After one course of preoperative chemotherapy (AI: doxorubicin, uromithexane, and ifomide), the patient achieved stable disease (SD). Because grade 4 hyponatremia occurred as an adverse effect of the therapy, the chemotherapy was terminated after one course, and the patient elected to undergo surgery.

The patient underwent an open subtotal esophagectomy with a right thoracotomy and an anterior chest wall stem jejunostomy with vascular reconstruction. The tumor was located in the cervicothoracic transition area, requiring the esophagus to be dissected near the esophagogastric junction. The patient was positioned in the supine position, and the abdomen was opened. The esophagus was withdrawn from the neck and was dissected 1 cm cranially from the tumor. Jejunal construction was performed with a pedicled flap. The second jejunal artery was dissected, and the jejunum was elevated in front of the chest wall for reconstruction. A rapid, intraoperative, pathological diagnosis denied any malignancy at the esophageal margin. The operation time was 8 h and 10 min, and the blood loss was 340 mL.

**Fig. 7:**
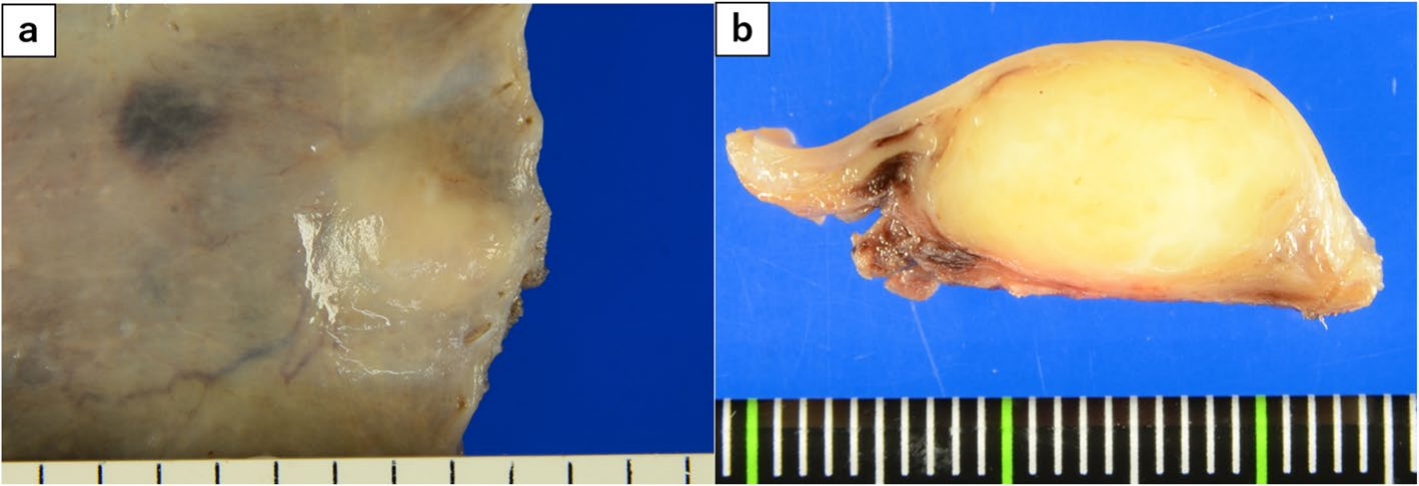
Fresh resection specimen. **a** A white, solid tumor with a 1.8-cm maximum diameter was observed. **b** The dissected surface was uniform, and no internal necrosis was evident

Figure 7 shows the fresh resection specimen. A white, solid tumor with a maximum diameter of 1.8 cm was observed in the cervicothoracic esophagus. The cut surface revealed a uniformly yellowish-white, solid mass centering on the muscularis propria and clearly bordering and compressing the surrounding area. No internal necrosis was found.

**Fig. 8:**
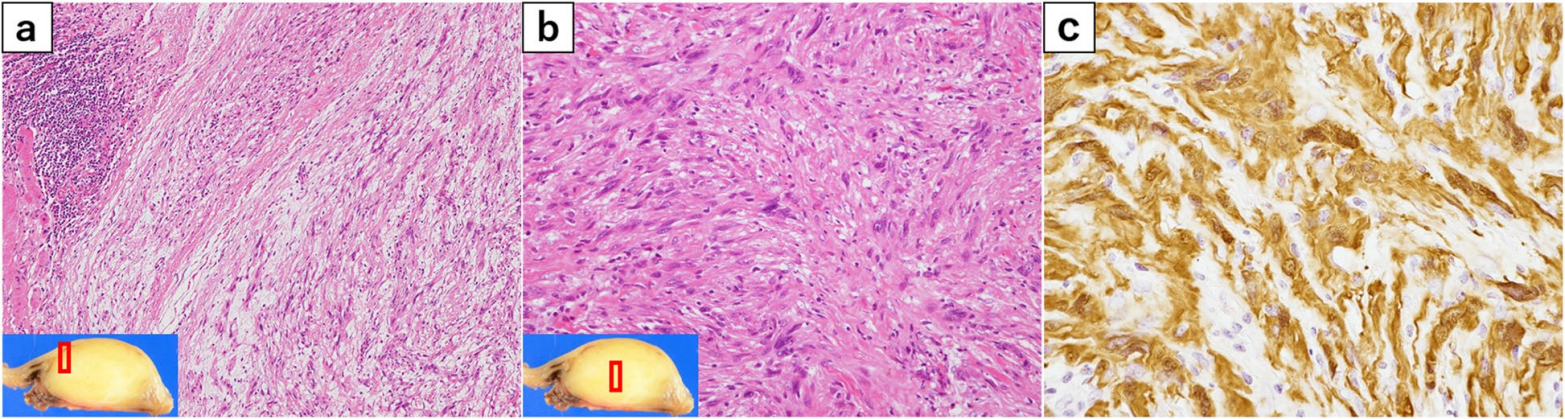
Histopathological findings. **a** Hematoxylin–eosin staining, × 100. The tumor was located within the intrinsic muscularis propria and contained a proliferation of spindle-shaped cells and surrounding lymphoid cuff characteristic of schwannoma. **b** Hematoxylin–eosin staining, × 200. The tumor center revealed increased cell density, mitotic figures, and nuclear atypia. **c** Immunohistochemical staining. Neurogenic marker S100 was positive

Histopathological analysis (Fig. 8) found that the tumor was located within the intrinsic muscularis propria. The tumor margins contained a proliferation of spindle-shaped cells and lymphoid cuff suggestive of schwannoma. On the other hand, the tumor center was sarcoma-like, with high cell density and a bundle-like or scattered cellular distribution. The nucleus of the centrally located spindle-shaped cells was spindled to round or mildly atypical with chromatin irregularities; only a few nuclei were large and severely atypical. Mitotic figures showed an increase to 1–2 cells/10 high power field (HPF). Immunohistochemically, the tumor cells stained diffusely positive for S100 protein and SOX10, indicating Schwann cell differentiation, and MIB1-positive cells showed an increase of up to 10% in the tumor center. Based on these features, low-grade MPNST was finally diagnosed.

## Discussion

MPNST is a malignant spindle cell tumor arising from the peripheral nerves anywhere in the body and accounts for 3–5% of soft tissue sarcoma cases [1]. About half the cases characteristically occur against a background of neurofibromatosis type 1 (NF1) while about 40% are sporadic, and 10% occur after irradiation [1, 2]. Age at onset is commonly 20–50 years, and the most common sites of onset are the soft tissues of the trunk and extremities [1, 3–5]. MPNSTs are highly aggressive tumors characterized by rapid growth with infiltration of surrounding tissue and hematogenous metastases [6].

Surgery is the first-line treatment. For non-infiltrative soft-tissue sarcomas, a surgical margin width greater than 5 mm decreases the risk of local failure regardless of the use of adjuvant radiotherapy [7]. Based on the literature review, the treatment recommendations for MPNST are as complete surgical removal is the strongest predictor of survival, every effort should be made to perform radical tumor excision with as wide a margin of normal tissue as is feasible to achieve free histologic margins [6].

Chemotherapy and radiation therapy may also be used. For chemotherapy, doxorubicin and ifosfamide, the chosen chemotherapy regimen for this tumor, are standard drugs used in the treatment of soft tissue sarcomas [8, 9]. Neoadjuvant chemotherapy for localized high-risk soft tissue sarcoma has been tested in several trials, showing a 5% to 10% overall survival (OS) benefit [10–12]. Doxorubicin plus ifosfamide regimen should choose whenever neoadjuvant chemotherapy is used in patients with high-risk soft tissue sarcoma [13, 14]. Radiotherapy has not been shown to be effective [15]. In general the prognosis of MPNST is extremely poor, with a 5-year survival rate of 34% and a 10-year survival rate of 23% [3]. Most distant metastases occur within two years, with the most common metastatic organ being the lung, followed by bone [4].

MPNST of esophageal origin is very rare, and to the best of our knowledge, there are only 16, previous case reports (eight in Japan) of MPNST of esophageal origin. We reviewed 17 cases, including the present one (Table 1) [16–25], and found that the age at onset ranged from 27 to 76 years and that female patients (*n* = 13) were more numerous than male patients (*n* = 4). The initial symptom was dysphagia in 13 patients; the remaining four patients were asymptomatic, their condition being detected by an imaging examination. The most common site of origin was the lower thoracic esophagus. The tumor diameter ranged from 1.8 cm to 17 cm. Sixteen patients received surgery, and one received chemoradiotherapy. MPNST was diagnosed before treatment in only five patients, but the condition was misdiagnosed as benign leiomyoma in five patients, indicating the difficulty of determining the malignancy status.

**Table 1:** Previous reports of esophageal MPNST

Year	Age	Sex	Nationality	Symptom	Size	Macroscopic feature	Preoperative diagnosis	Metastasis	Treatment	Prognosis
1993^5)^	56	F	Japan	Abnormal shadow on CXR	4.8*4.2*3.0	SMT	Leiomyoma	-	Enucleation	Alive(28month)
1996^6)^	57	F	Japan	Dysphagia	4.0*3.5*2.7	SMT	Not available	-	Enucleation	Alive(24month)
2000^7)^	60	F	Germany	Dysphagia, general fatigue, appetite loss, body weight loss	17	SMT	MPNST	-	Oesophagectomy	Alive(48month)
2001^8)^	49	F	Japan	None	8.2*5.8*3.7	SMT	Leiomyoma	-	Enucleation	Alive(27month)
2003^9)^	55	M	Japan	Dysphagia	8.5*7.0*4.0	SMT	Submucosal tumor	-	Enucleation	Alive(20month)
2003^6)^	49	F	Japan	Dysphagia, cough	8.2*5.8*3.7	SMT	Leiomyoma	-	Enucleation	n/a
2004^10)^	54	M	Spain	Dysphagia	6(gastroscopy)	Ulcer	MPNST	-	Oesophagectomy	Alive(2month)
2006^11)^	54	F	Turkey	Dysphagia, mass in neck	6*6	SMT	Not available	-	Oesophagectomy	Alive(40month)
2010^6)^	20	F	Russia	Dysphagia	8.8*3.0*3.5	SMT	Not available	-	Enucleation	Alive(36month)
2010^12)^	62	M	Japan	Dysphagia, fever, body weight loss	Unknown	Irregularity	Non-small cell undifferentiated esophageal cancer	Lymph nodes	Chemo-radiation	Death(12month)
2011^13)^	44	F	China	Dysphagia	5.5*4.0*4.5	SMT	Leiomyoma	-	Enucleation	Alive(72month)
2012^14)^	64	F	Japan	Abnormal shadow on CXR	8.0*7.5*4.0	SMT	MPNST	-	Oesophagectomy	Alive(24month)
2012^6)^	43	M	Singapore	Dysphagia	4.5*4.0*2.5	SMT	Not available	-	Enucleation	Alive(12month)
2016^15)^	27	F	India	Dysphagia, palpitation, body weight loss	12*10*10	SMT	Leiomyoma	-	Oesophagectomy	Alive(18month)
2017^16)^	76	F	Italy	Dysphagia	3.9	ulcer	Malignant tumor	Left adrenal gland, ribs, vertebras	Oesophagectomy, chemotherapy	Death(5month)
2018^6)^	31	F	Japan	Dysphagia	7.1*6.1*5.5	Protruding type with tumor	MPNST	-	Oesophagectomy	Alive(16month)
2021	67	F	Japan	Abnormality on CT	1.8*1.7*1.5	SMT	MPNST	-	Oesophagectomy	Alive(4month)

MPNST often occurs in the context of NF1 as a secondary, malignant transformation of multiple benign neurofibromas throughout the body. The present case was rare and exceptional in that it did not occur against a background of NF1, had weak malignancy, and had a schwannoma-like component in the surrounding tissue. The latest WHO classification of bone and soft tissue tumors (5th edition) [1] clearly defines MPNST occurring in NF1 as intermediate between a benign and malignant tumor. On the other hand, malignant transformation of a schwannoma into MPNST is quite exceptional, and there is as of yet no classification of the borderline areas or much prognostic data. In the present case as well, determining the malignancy status of the lesion by analyzing the biopsy specimen and diagnosing MPNST were difficult. However, the CT findings of the remarkable increase in tumor size over a short period of time, together with the other findings, led conclusively to the diagnosis of MPNST.

The pathomechanism of primary MPNST of the esophagus in the 17 cases reviewed demonstrated no association with NF1 or radiotherapy. Moreover, no evidence of a schwannoma-like component or low-grade MPNST was found. Interestingly, in the present case, the sarcoma developed within an area irradiated 15 years previously for the treatment of left breast cancer. The average latency period of sarcomas is approximately 11 years after irradiation [26]. The pathology of the present case suggests the possibility of a malignant transformation from schwannoma to MPNST, which is extremely rare. The site of the MPNST in the esophagus that occurred in this case and the irradiated area just barely coincide. It may be more likely that irradiation contributed to the malignant transformation of an existing, esophageal schwannoma than that it caused de novo MPNST.

We reported a very rare case of esophageal MPNST which was treated by resection. The diagnosis was based not only on pathological findings, but also on the rapid growth of the lesion over a short period of time.

## Data Availability

Not applicable.

## Abbreviations

MPNST: Malignant peripheral nerve sheath tumor
CT: Computed tomography
NF1: Neurofibromatosis type 1
FNA: Fine needle aspiration
EUS-FNA: Endoscopic ultrasound-guided fine needle aspiration
PET-CT: Positron emission tomography-computed tomography
SD: Stable disease
HPF: High power field
OS: Overall survival
